# Efficacy of Platelet-Rich Plasma in the Treatment of Chronic Plantar Fasciitis Compared with Other Conventional Treatments (Corticosteroids and Shockwave Therapy): A Systematic Review

**DOI:** 10.3390/ijms27219550

**Published:** 2026-10-26

**Authors:** Mercedes Ortiz-Romero, Elías Parra-Aguado, Luis María Gordillo-Fernández

**Affiliations:** 1 Department of Podiatry, Faculty of Nursing, Physiotherapy and Podiatry, University of Seville, 41009 Seville, Spain; mortiz17@us.es (M.O.-R.); lgordillo@us.es (L.M.G.-F.); 2 Research Group “Biomedical Bases of the Foot Affecting Support and Gait”, Institute of Biomedicine of Seville (IBiS), 41013 Seville, Spain; 3 Faculty of Nursing, Physiotherapy and Podiatry, University of Seville, 41003 Seville, Spain

**Keywords:** plantar fasciopathy, heel pain, autologous biological therapy, extracellular matrix, inflammatory mediators, growth factors, ultrasonography

## Abstract

Plantar fasciitis is a degenerative condition of the plantar fascia characterized by collagen fiber degeneration, increased ground-substance (extracellular matrix) proteins, focal fibroblast proliferation and increased vascularity; it is for ≥3 months and affects around 10% of the general population. This review compared the efficacy of platelet-rich plasma (PRP), an autologous source of growth factors, with corticosteroid (CS) injection, which acts through anti-inflammatory mechanisms, and extracorporeal shockwave therapy (ESWT), which acts through mechanotransduction. Following a protocol registered retrospectively in Open Science Framework (OSF) Registries, PubMed/MEDLINE, Scopus, Web of Science, Cochrane Library and Dialnet were searched from January 2020 to September 2026 for comparative studies of PRP versus CSs and/or ESWT in adults with chronic plantar fasciitis. Screening was performed independently by two reviewers. Because of heterogeneous outcome scales, time points and reporting, pooling was not appropriate and results were synthesized narratively by comparator and follow-up period. Twenty-two primary studies (1915 patients) were included: 20 compared PRP with CSs and two compared PRP with ESWT. Short-term (≤6 weeks) results for PRP versus CSs were inconsistent, with some studies favoring CSs. From approximately 3 months onward, most studies reported lower pain (visual analogue scale), better function (American Orthopaedic Foot & Ankle Society (AOFAS), Foot and Ankle Ability Measure (FAAM), Foot Function Index (FFI)) and a greater reduction of ultrasonographic plantar fascia thickness with PRP; three studies found no difference or favored CSs. The two studies comparing PRP with ESWT reported mixed results. The available evidence suggests that PRP may provide greater medium- to long-term improvements in pain and function than CS injection, whereas short-term results are inconsistent. Evidence comparing PRP with ESWT is insufficient to draw conclusions. According to Grading of Recommendations Assessment, Development and Evaluation (GRADE), the certainty of the evidence was low for long-term pain and plantar fascia thickness and very low for the remaining outcomes and for the comparison with ESWT.

## Simple Summary

Plantar fasciitis is the most common cause of heel pain and affects around 10% of the population. When pain lasts more than three months, it is considered chronic and is often difficult to treat. This review brought together recent studies (2020–2026) comparing platelet-rich plasma, a preparation obtained from the patient’s own blood, with two widely used treatments: corticosteroid injections and shockwave therapy. In several studies, corticosteroids relieved pain faster during the first weeks, although short-term results were not consistent, and this benefit did not last. From about three months onward, platelet-rich plasma led to less pain, better foot function and a greater reduction in the thickness of the plantar fascia measured by ultrasound. Only two studies compared it with shockwave therapy, so that comparison remains uncertain. These findings may help clinicians choose a treatment depending on whether a patient needs rapid relief or longer-lasting improvement.

## 1. Introduction

The plantar fascia is a dense, regularly arranged connective structure that originates from the calcaneal tuberosity and divides into medial, central and lateral bands, the central band being the largest, thickest and most resistant [1,2]. It is composed mainly of type I collagen fibers arranged in a proximal–distal direction; where the fiber bundles change direction, type III collagen and loose connective tissue rich in hyaluronan allow the bundles to glide (Table 1) [3]. The fascia contains free nerve endings and Ruffini and Pacini corpuscles, giving it a proprioceptive role [2,3]. Although it is surrounded by a perifascial vascular plexus, it is a hypovascular structure, with a critical zone of lowest perfusion 1–2 cm from its proximal insertion that predisposes it to degenerative disorders [2].

Functionally, the plantar fascia supports and maintains the medial longitudinal arch and participates in two biomechanical mechanisms [4,5]. In the windlass mechanism, dorsiflexion of the toes, especially the hallux, winds the fascia around the proximal phalanges, shortening the distance between its origin and insertion, raising the arch and stiffening the foot for propulsion [4]. In addition, the fascia acts as an elastic spring: when the foot is loaded during stance, the arch is compressed and the foot lengthens, stretching the fascia, which stores elastic energy and returns most of it when the foot is unloaded, assisting push-off [4].

Plantar fasciitis refers to a disorder of the plantar fascia characterized by deterioration of collagen fibers, increased secretion of ground-substance proteins, focal areas of fibroblast proliferation and increased vascularization [6,7]; it is considered chronic when symptoms persist for ≥3 months [6,7]. When the affected tissue shows minimal or no inflammatory cells and a progressive degenerative process predominates, the condition is termed plantar fasciosis [8,9,10]. In fact, histopathological studies have not shown clear evidence of an inflammatory process in chronic cases, but rather degenerative changes, which has led to the broader term plantar fasciopathy and questions the rationale for purely anti-inflammatory treatments [11].

Plantar fasciopathy is the most common cause of heel pain in ambulatory care. It affects around 10% of the general population, approximately 790 million people worldwide, with a peak prevalence between 45 and 64 years and up to 17.5% in runners, and it entails a substantial economic burden [6,9]. Its etiology is multifactorial and is attributed to repetitive microtrauma of the fascia, favored by intrinsic factors (female sex, age, body mass index > 25 kg/m^2^, flat or cavus foot, gastrocnemius–soleus shortening, leg-length discrepancy, heel fat-pad atrophy, reduced ankle dorsiflexion, increased subtalar pronation and increased tibial or femoral torsion) and extrinsic factors (footwear, activity level, trauma and surface properties) [6,12,13,14]. Clinically, it presents with post-static dyskinesia: sharp pain at the inferomedial heel, at the origin of the fascia, during the first steps in the morning or after rest, which decreases with walking but may worsen after prolonged standing or excessive activity [6,15]. Diagnosis is primarily clinical, by reproducing pain on palpation of fascial origin with passive toe extension [15]. When the diagnosis is uncertain or pain persists for 4–6 months, imaging is recommended [12]; ultrasonography is the most effective and cost-effective test, with supportive findings including a plantar fascia thickness (PFT) > 4 mm, hypoechoic areas, perifascial fluid and loss of the fibrillar pattern [13,16].

Numerous treatments are available for this condition [12,13]. Local corticosteroid (CS) injection is one of the most widely used: synthetic glucocorticoids exert anti-inflammatory effects that provide rapid pain relief, but their use has been associated with complications such as heel fat-pad atrophy and plantar fascia rupture [11]. Extracorporeal shockwave therapy (ESWT) delivers short, high-energy acoustic pulses that propagate through tissue and induce biological effects through mechanotransduction. Its mechanism of action is not fully understood; four phases have been proposed—physical (extracellular cavitation, increased membrane permeability and ionization), physicochemical (diffusion of free radicals), chemical (intracellular reactions and molecular changes) and biological—leading to anti-inflammatory, analgesic and neovascular effects, increased release of growth factors and stimulation of tissue regeneration [17].

Platelet-rich plasma (PRP) is an autologous biological therapy: it is obtained from the patient’s own venous blood, collected with an anticoagulant and centrifuged once or twice to concentrate platelets in the plasma fraction, which can then be activated chemically (e.g., calcium chloride or thrombin) or physically, or injected non-activated into soft tissues [18,19]. After appropriate preparation, it can reach a platelet concentration up to five times higher than the baseline values of a healthy individual (150,000–400,000 platelets/μL) [19,20]. Platelet alpha granules release cytokines and growth factors—such as transforming growth factor β (TGF-β), platelet-derived growth factor (PDGF), epidermal growth factor (EGF), fibroblast growth factor (FGF), insulin-like growth factor 1 (IGF-1), hepatocyte growth factor (HGF) and stromal cell-derived factor 1α (SDF-1α)—that regulate cell proliferation and differentiation, chemotaxis, collagen homeostasis and angiogenesis [18]. Accordingly, its main proposed actions are modulation of inflammation, acceleration of soft-tissue regeneration and repair, and stimulation of angiogenesis [18,21].

However, no standardized protocol exists for the preparation and activation of PRP [18], and most previous meta-analyses have addressed a single comparison, such as PRP versus CSs [22,23] or CSs versus other interventions [11,24], whereas recent meta-analyses comparing PRP with ESWT or with several modalities pooled randomized trials only [25,26]. The novelty of the present review is that it updates this evidence with the most recent comparative studies (2020–2026), nine of which were published in 2025–2026, and brings together PRP versus both CSs and ESWT in a single PRISMA-based synthesis, focusing on the time course of the response across pain, function and ultrasonographic PFT in order to derive indications for each treatment. Therefore, the aim of this systematic review was to compare the efficacy of PRP with that of CSs and ESWT in chronic plantar fasciitis in terms of short-, medium- and long-term pain, function and PFT.

## 2. Methods

### 2.1. Protocol, Registration and Ethics

This systematic review was conducted and reported in accordance with the Preferred Reporting Items for Systematic Reviews and Meta-Analyses (PRISMA) 2020 statement [27]. The protocol was registered retrospectively in OSF Registries (https://osf.io/hdcjb, accessed on 20 September 2026) before the updated search, screening and data extraction were performed. Ethical approval was not required because this study is a systematic review of previously published data and did not involve new data collection from human participants or animals.

### 2.2. Eligibility Criteria

Eligibility was defined according to the PICOS framework. Inclusion criteria:

Population: adults (≥18 years) with a clinical diagnosis of chronic plantar fasciopathy (symptoms ≥ 3 months), with or without prior conservative treatment.

Intervention: local injection of autologous PRP, any preparation, number of injections or guidance method.

Comparator: local CS injection and/or ESWT.

Outcomes: pain (VAS) as the primary outcome; function (AOFAS, FFI, FAAM), PFT, adverse events or recurrence as secondary outcomes.

Study design: RCTs (main analysis) or non-randomized controlled studies with a concurrent comparator (separate analysis).

Full-text articles published from January 2020 to the search date.

Exclusion criteria:

Heel pain of other origin, acute cases or previous plantar fascia surgery.

Studies in which PRP was not one of the interventions, or in which PRP was combined with another active co-intervention not received by the comparator group.

Studies without an eligible comparator.

Single-arm studies, case series, case reports, narrative and systematic reviews, letters, editorials, conference abstracts, and animal or cadaveric studies.

Systematic reviews and meta-analyses retrieved by the search were not included in the synthesis; they were used only to contextualize the findings in Section 3.5.

### 2.3. Information Sources and Search Strategy

Five databases were searched on 20–21 September 2026: PubMed/MEDLINE, Scopus, Web of Science Core Collection, Cochrane Library and Dialnet, for records published from January 2020 to the search date. The search strings followed a PICOS-based structure combining controlled vocabulary (MeSH) and free-text terms for the condition, PRP and the comparators (CSs and ESWT); the full strings for each database, with the exact search date, are provided in Supplementary Table S1 and in the registered protocol. The search was limited to studies published from January 2020 onward in order to update the evidence synthesized in previous systematic reviews and meta-analyses, which included earlier trials [22,24]. Consequently, the present review should be regarded as an update of the recent evidence rather than a comprehensive synthesis of all published trials, and its findings are compared with those earlier reviews in Section 3.5.

### 2.4. Study Selection

**Figure 1:**
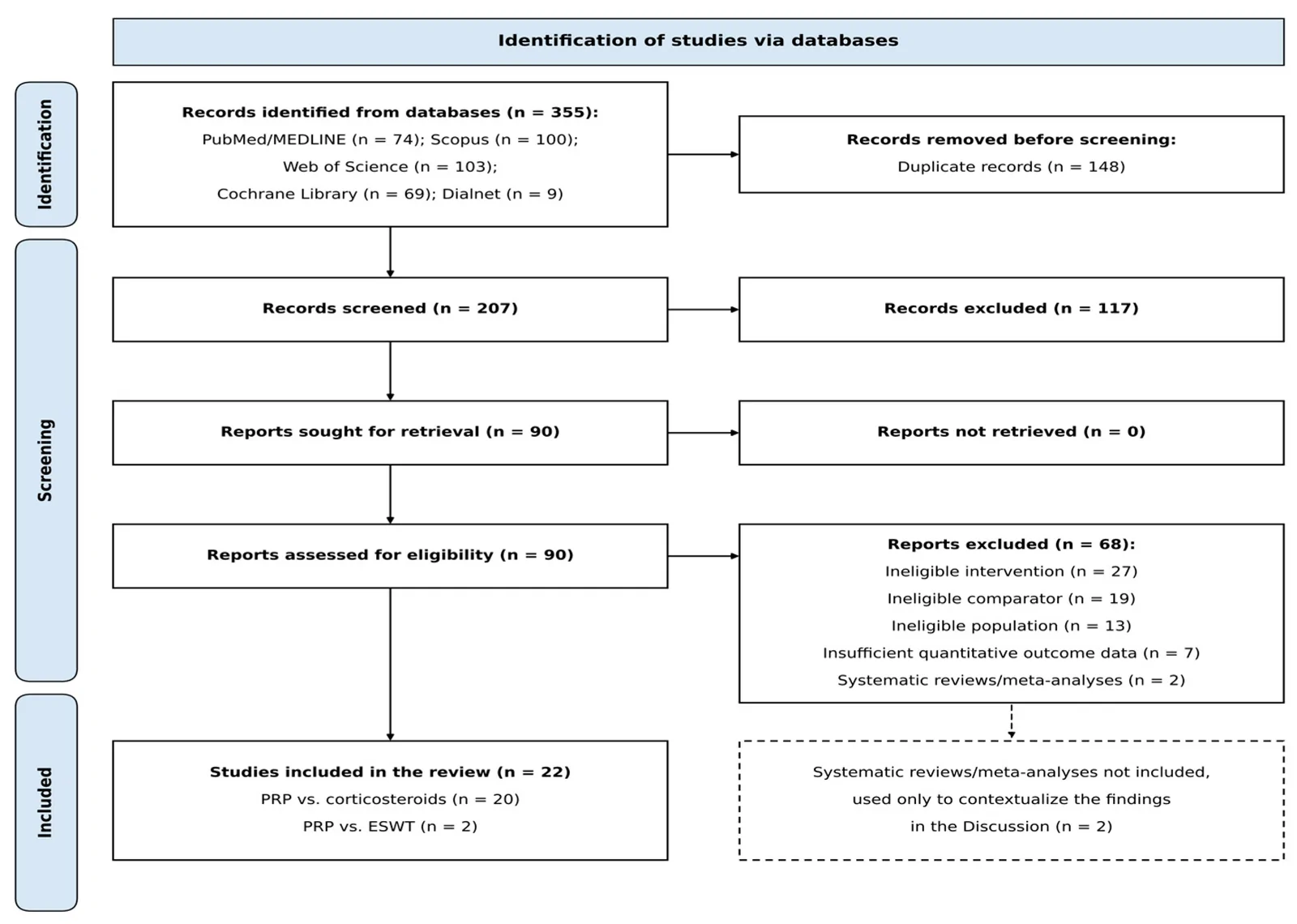
PRISMA 2020 flow diagram of the study selection process.

Records were imported into Zotero (ver. 10.0.6, Corporation for Digital Scholarship, Vienna, VA, USA) and deduplicated. Title and abstract screening was performed independently and in a blinded manner by two reviewers using Rayyan (web version: Rayyan System Inc. Cambridge, MA, USA) [28], after a calibration pilot on 50 randomly selected records. Records selected by either reviewer proceeded to full-text assessment; disagreements were resolved by discussion and, where necessary, by a third reviewer. The selection process is shown in Figure 1.

A total of 355 records were identified: PubMed/MEDLINE (*n* = 74), Scopus (*n* = 100), Web of Science Core Collection (*n* = 103), Cochrane Library (*n* = 69) and Dialnet (*n* = 9). After removal of 148 duplicates, 207 records were screened by title and abstract, of which 117 were excluded. Ninety full-text articles were assessed for eligibility and 68 were excluded, including two systematic reviews with meta-analysis [25,26], which did not meet the predefined design criteria and are therefore considered only in Section 3.5. The remaining 66 reports were excluded because of an ineligible intervention (*n* = 27), an ineligible comparator (*n* = 19), an ineligible population (*n* = 13) or insufficient quantitative outcome data (*n* = 7). Finally, 22 primary studies were included (Figure 1).

### 2.5. Data Extraction and Synthesis

Data on study design, sample size, comparison, outcome measures, follow-up time points and results (means, standard deviations and *p*-values as reported by the original authors) were extracted using a standardized form. A quantitative meta-analysis was not considered appropriate because the studies used different outcome scales and follow-up time points, eight studies reported their results only in summary form without means and standard deviations, and PRP, CS and ESWT protocols varied substantially. Results were therefore synthesized narratively, grouped by comparison (PRP vs. CSs; PRP vs. ESWT), outcome (pain, function, PFT and adverse events) and follow-up period, defined as short term (≤6 weeks), medium term (>6 weeks to <6 months, typically 3 months) and long term (≥6 months).

### 2.6. Certainty of the Evidence

The certainty of the evidence was rated for each comparison, outcome and follow-up period using the Grading of Recommendations Assessment, Development and Evaluation (GRADE) approach [29], adapted for syntheses without a single pooled effect estimate [30]. Bodies of evidence consisting predominantly of RCTs started as high certainty and those based on non-randomized studies as low certainty, and were downgraded for risk of bias, inconsistency, indirectness, imprecision and publication bias. Certainty was classified as high, moderate, low or very low and is presented in a summary of findings table.

## 3. Results and Discussion

### 3.1. Characteristics of the Included Studies

The 22 included studies (Table 2) comprised 13 randomized controlled trials (RCTs) and 9 non-randomized comparative studies, with a total of 1915 patients (range 32–230 per study). Twenty studies (1788 patients) compared PRP with CSs and two (127 patients) compared PRP with ESWT. Pain was assessed with the visual analog scale (VAS) in all 22 studies; function with the American Orthopaedic Foot & Ankle Society (AOFAS) score in 13, the Foot Function Index (FFI) in four and the Foot and Ankle Ability Measure (FAAM) in three; and PFT in four. Follow-up ranged from 15 days to 2 years, and 19 of the 22 studies had a final follow-up of 6 months (24 weeks). Study selection was complete at the time of the search.

Table 3 summarizes the symptom duration criteria and the intervention protocols reported by the included studies. Regarding the chronicity criterion (≥3 months), it was met in 12 studies [33,34,35,37,38,41,42,43,45,46,50,51]. Seven studies used shorter thresholds: heel pain of more than 4 weeks [32] or 6 weeks [39,47,48], symptoms of more than 2 months [36], or failure of at least 2 months [31] or 6 weeks [52] of conservative treatment; in the latter, 60–70% of patients had symptoms for more than 6 months. Three studies did not state a criterion [40,44,49], although in Batra et al. [44] the mean symptom duration was approximately 8 months. The PRP protocols differed in blood volume, centrifugation method (single, double or triple spin), volume obtained or injected (2–5 mL), number of injections (one or three) and image guidance (ultrasound or palpation guided), and three studies did not report the PRP preparation [42,44,49]. Only one study reported the platelet concentration relative to baseline [52], and none reported leukocyte content or activation. Most CS protocols used methylprednisolone, mostly at 40–80 mg, whereas one study used triamcinolone acetonide [45].

### 3.2. PRP Versus CSs

#### 3.2.1. Pain

Short-term (≤6 weeks) results were inconsistent (Table 4). Prakash and Kumar [37] found lower pain with CSs at 6 weeks (4.86 ± 1.02 vs. 7.05 ± 0.72) and 12 weeks (4.08 ± 0.72 vs. 6.34 ± 0.82) and Yadav et al. [52] at 6 weeks (1.50 ± 0.73 vs. 4.80 ± 0.93; 79.9% vs. 37.4% improvement from baseline) and 12 weeks (2.13 ± 1.04 vs. 3.33 ± 1.15). Sathyendra et al. [38] and Kumar K. et al. [41] found no difference at 15 days (7.03 ± 0.79 vs. 7.09 ± 0.98; *p* = 0.883, in the latter). In contrast, Khurana et al. [32] reported lower pain with PRP at 2 weeks (6.71 ± 0.99 vs. 7.30 ± 1.09) and 4 weeks (3.98 ± 1.03 vs. 4.93 ± 1.07), and Shah et al. [49] reported lower pain with PRP from the earliest assessments at 2 weeks (5.53 ± 0.776 vs. 6.47 ± 0.937; *p* < 0.001) and 4 weeks (4.70 ± 0.466 vs. 5.27 ± 0.868; *p* = 0.003).

From 1 month onward, most studies favored PRP. Kumar K. et al. [41] observed lower VAS scores with PRP at 1 month (6.26 ± 0.7 vs. 6.69 ± 0.87; *p* = 0.035), 3 months (5.37 ± 0.69 vs. 6.2 ± 0.72; *p* < 0.0001) and 6 months (3.71 ± 0.67 vs. 5.4 ± 0.65; *p* < 0.0001); Sathyendra et al. [38] reported significantly greater relief with PRP from 1 to 6 months; Shah et al. [49] found lower pain with PRP at 12 weeks (2.10 ± 0.712 vs. 2.77 ± 0.858; *p* = 0.002); and Kamala and Hanumantharaya [40] reported significant relief with PRP from 6 weeks, maintained at 3 and 6 months, with significantly greater improvements than with CSs.

At 6 months (24 weeks), VAS scores were lower with PRP in Khurana et al. [32] (0.52 ± 0.60 vs. 1.92 ± 1.03; *p* < 0.001), Prakash and Kumar [37] (2.9 ± 1.07 vs. 4.46 ± 0.96; *p* < 0.0001), Sharma R. et al. [39] (1.97 ± 1.13 vs. 2.71 ± 0.94), Yadav et al. [52] (1.43 ± 0.57 vs. 3.23 ± 1.01; 81.4% vs. 56.8% improvement, with some deterioration in the CS group between 12 and 24 weeks) and Priyam et al. [42] (1.9 ± 0.8 vs. 2.5 ± 0.9), in the largest sample of this subgroup (230 patients). Vellingiri et al. [34] also reported PRP superiority at 6 months. A further eight studies [43,44,45,46,47,48,50,51], reported only in summary form, described an early CS advantage followed by PRP superiority at medium-to-long term.

#### 3.2.2. Function

Functional outcomes followed the same pattern. Khurana et al. [32] reported a 6-month AOFAS score of 95.09 ± 4.60 with PRP versus 85.96 ± 5.34 with CSs (*p* < 0.001). In Prakash and Kumar [37], PRP surpassed CSs from 12 weeks (82.38 ± 2.26 vs. 78.62 ± 2.48), and the difference widened at 6 months (86.64 ± 1.48 vs. 75.15 ± 2.02). Sharma R. et al. [39] found a 6-month AOFAS of 86.04 ± 7.45 versus 81.23 ± 9.60 and Shah et al. [49] a significantly higher FAAM with PRP at 12 weeks (91.03 ± 3.943 vs. 86.13 ± 6.388; *p* = 0.001).

#### 3.2.3. Plantar Fascia Thickness

PFT decreased more markedly with PRP than with CSs in Prakash and Kumar [37] (from 5.76 ± 0.65 to 3.32 ± 0.44 mm vs. from 5.65 ± 0.62 to 3.73 ± 0.65 mm; *p* = 0.007) and was lower with PRP at 6 months in Sharma R. et al. [39] (3.53 ± 0.81 vs. 4.58 ± 1.02 mm).

#### 3.2.4. Satisfaction and Adverse Events

Priyam et al. [42] reported 85% patient satisfaction with PRP versus 65% with CSs. Vellingiri et al. [34] observed complications in two patients treated with PRP (superficial infection) versus 10 treated with CSs (five superficial infections, three cases of skin depigmentation and two of fat-pad atrophy).

#### 3.2.5. Discordant Results

Not all studies supported PRP superiority. Tabrizi et al. [31], in obese patients, found significantly better results in the CS group (*p* < 0.001), and Kumar N. [33] found no significant difference between treatments at any follow-up point.

### 3.3. PRP Versus ESWT

Pandey et al. [36], in an RCT of 72 patients, compared ESWT with PRP using the VAS, AOFAS and ultrasonographic PFT, with follow-up at days 15, 30 and 90. Baseline VAS scores were comparable (ESWT 6.44 ± 1.11 vs. PRP 6.78 ± 1.17; *p* = 0.237). At day 15, the ESWT group had lower pain (4.67 ± 1.45 vs. 6.67 ± 1.35; *p* < 0.001); at day 30 both groups were comparable (4.97 ± 1.46 vs. 4.69 ± 1.39; *p* = 0.391); and by day 90, PRP showed clearly lower pain (3.36 ± 0.96 vs. 5.47 ± 1.63; *p* < 0.001), with a similar pattern for PFT and AOFAS. Alessio-Mazzola et al. [35], in a retrospective study of 55 patients (24 athletes) with a minimum follow-up of 2 years, found no significant differences between ultrasound-guided PRP and focal ESWT in VAS and FFI, although the ESWT group had a higher, non-significant failure rate and required more follow-up visits, and athletes treated with PRP returned to sport significantly faster (*p* = 0.044).

### 3.4. Certainty of the Evidence

The certainty of the evidence according to GRADE is summarized in Table 5. For PRP versus CSs, it was low for long-term pain and for PFT, and very low for short-term and medium-term pain, function and adverse events. For PRP versus ESWT, the certainty of the evidence was very low.

Evidence from bodies of predominantly randomized studies started as high certainty, and that from non-randomized studies as low certainty. Risk of bias was rated serious owing to non-randomized and retrospective designs, small samples and results reported only in summary form. Imprecision was rated serious because no pooled estimate could be calculated and samples were small. Indirectness was not rated as serious, although the ≥3-month chronicity criterion could not be confirmed in several studies (Table 3). Publication bias was suspected because of the predominance of small positive studies. CSs, corticosteroids; ESWT, extracorporeal shockwave therapy; PFT, plantar fascia thickness; PRP, platelet-rich plasma; RCT, randomized controlled trial.

### 3.5. Interpretation of the Findings and Comparison with Previous Reviews

In the comparison between PRP and CSs, which is supported by 20 of the 22 included studies, a consistent time-dependent pattern was observed in the medium and long term: from approximately 3 months onward, most studies reported lower pain, better function and a greater reduction of PFT with PRP. In the short term, results were inconsistent: some studies favored CSs, two found no difference at 15 days and two favored PRP from 2 weeks. The evidence comparing PRP with ESWT is considerably more limited, as it relies on only two primary studies with mixed results. The two evidence bases are therefore not equivalent, and conclusions regarding PRP versus ESWT should be regarded as preliminary.

These findings may be related to the different mechanisms of action of each treatment, although these mechanisms were not assessed in the included studies. CSs act through anti-inflammatory mechanisms, which may explain the early analgesic effect observed in some studies; however, histopathological studies have not demonstrated a clear inflammatory process in chronic plantar fasciopathy, but rather degenerative changes [11], which may explain why their benefit is not sustained. PRP, in contrast, delivers growth factors that stimulate cell proliferation, collagen homeostasis and angiogenesis [18], which provides a biological rationale for a slower but more sustained effect. However, none of the included studies directly assessed tissue repair: the structural outcome measured was ultrasonographic PFT, and the greater reduction in PFT observed with PRP [37,39] should be interpreted as an imaging finding rather than as evidence of tissue repair.

These findings are broadly in agreement with those of previous systematic reviews and meta-analyses. Fei et al. [22] concluded that PRP was more effective than CSs in relieving pain and improving foot and ankle function at mid-term follow-up, without significant functional differences at short term. Seth et al. [24] reported better VAS scores with PRP than with CSs at 3 and 6 months, whereas differences in the AOFAS score appeared only at 6 months; they also found ESWT superior to CSs at 6 months. Aleid et al. [23], in 599 patients, concluded that PRP is promising for improving function and may provide greater pain relief than CSs, and that the functional benefits of CSs are not maintained over time. Peña-Martínez et al. [11], in 1109 patients, found that CS injections did not offer better results than other common interventions in reducing PFT or relieving pain.

Regarding ESWT, Pandey et al. [36] observed an early advantage of ESWT at day 15, which may reflect its analgesic effect [17], followed by an advantage of PRP at day 90, whereas the retrospective study by Alessio-Mazzola et al. [35] found no significant differences in its main overall outcome measures at a minimum of 2 years, although athletes treated with PRP returned to sport significantly earlier. Two meta-analyses of RCTs, which were not included in the present synthesis, provide additional context. Daher et al. [25] (6 RCTs; 214 PRP and 218 ESWT patients) found a statistically greater improvement with PRP in VAS (mean difference −0.67; 95% CI −1.16 to −0.18; *p* = 0.007) and PFT (mean difference −0.56; 95% CI −0.77 to −0.35; *p* < 0.001), although the pain difference did not reach clinical significance. Herber et al. [26] (21 RCTs; 1356 patients) found a significantly greater VAS improvement with PRP than with ESWT (standardized mean difference 0.86; 95% CI 0.30 to 1.41; *p* = 0.002); PRP also improved the AOFAS score more than CSs, but not more than ESWT, and no significant differences were found among PRP, CSs and ESWT in FFI or in the reduction of PFT. This last finding contrasts with the primary studies [37,39] and with Daher et al. [25], so PFT results should be interpreted with caution.

The discordant results also deserve comment. The CS advantage reported by Tabrizi et al. [31] was observed in obese patients, and a body mass index >25 kg/m^2^ is a recognized risk factor for plantar fasciopathy [6]; whether body weight modifies the response to PRP warrants specific investigation. In terms of safety, fewer local complications were reported with PRP than with CSs [34], consistent with the fat-pad atrophy and fascia rupture described after CS injection [11].

An important consideration is that PRP is not a uniform intervention. Platelet concentration, leukocyte content (leukocyte-rich or leukocyte-poor PRP), injected volume, activation method, number of and interval between injections, preparation method and the use of ultrasound guidance vary widely across protocols [18,19], and these characteristics were not analyzed in the present review. Consequently, the findings cannot be attributed to a specific PRP formulation or administration protocol, which limits their direct clinical applicability.

With these caveats, and given the low certainty of the evidence, PRP may be considered for patients with chronic plantar fasciopathy in whom conservative treatment has failed and who seek sustained medium- to long-term improvement. The position of ESWT relative to PRP cannot be established with the current evidence.

### 3.6. Limitations and Recommendations

This review has several limitations. The included studies differed in PRP preparation and administration, in the CS agent and dose and in ESWT parameters (density, frequency and number of sessions), and many did not report these characteristics in detail (Table 3); they were heterogeneous in sample size, outcome scales and follow-up time points, which precluded quantitative pooling; no formal study-level risk-of-bias assessment with a validated tool (RoB 2 or ROBINS-I) was performed, so risk of bias was considered only within the GRADE rating; the absence of pooling also precluded forest plots and statistical assessment of heterogeneity and publication bias; only two primary studies compared PRP with ESWT, one of them retrospective; eight studies were described only in summary form; the search was restricted to studies published from 2020 onward, so earlier trials were not included in the synthesis and the findings complement, rather than replace those of earlier reviews; the chronicity criterion (≥3 months) could not be confirmed in 10 of the 22 studies, as several used shorter thresholds (Table 3), although excluding them would not change the direction of the long-term findings for PRP versus CSs; the certainty of the evidence was low or very low (Table 5); and the protocol was registered retrospectively.

Several recommendations for future research derive from these limitations. First, adequately powered, multicenter RCTs with prospective registration, blinded outcome assessment and intention-to-treat analysis are needed, particularly for the comparison between PRP and ESWT. Second, studies should include only patients with symptoms for at least 3 months and report the mean symptom duration and previous treatments. Third, PRP should be described in detail—blood volume, centrifugation protocol, platelet concentration, leukocyte content, activation, injected volume, number of and interval between injections, and image guidance—together with the CS agent and dose and the ESWT device, energy flux density, number of pulses and sessions. Fourth, a core set of outcomes should be used, including the VAS, a validated foot function score and ultrasonographic PFT, with means and standard deviations reported at predefined time points (6 weeks, 3, 6 and 12 months) to allow future meta-analyses. Finally, subgroups that may respond differently, such as obese patients and athletes, deserve specific evaluation, as do cost-effectiveness and long-term recurrence.

For clinical practice, and considering the low certainty of the evidence, PRP may be considered in patients with chronic plantar fasciopathy who have not responded to conservative treatment and who seek medium- to long-term improvement, after informing them that short-term relief may be slower than with CSs in some cases. Clinicians are encouraged to use ultrasound for diagnosis and follow-up and to document the PRP preparation used, so that clinical experience can be compared across settings.

## 4. Conclusions

For PRP versus CSs (20 studies, 12 of them RCTs), PRP was associated with lower pain, better function and a greater reduction in ultrasonographic plantar fascia thickness from approximately 3 months onward, whereas short-term (≤6 weeks) results were inconsistent, with some studies favoring CSs. For PRP versus ESWT (two studies, one retrospective), the evidence is insufficient to draw conclusions. Because the certainty of the evidence was low for long-term pain and plantar fascia thickness and very low for the other outcomes, and PRP protocols varied widely, these findings should be interpreted with caution and confirmed in well-designed trials with standardized protocols and long-term follow-up.

## Figures and Tables

**Table 1:** Main components of the plantar fascia extracellular matrix and their function.

Component	Location and Function
Type I collagen	Main structural component; fibers arranged mainly in a proximal–distal direction, providing tensile resistance [3].
Type III collagen	Found where fiber bundles change direction (transverse and oblique fibers) [3].
Loose connective tissue and hyaluronan	Located between fiber bundles; allows them to glide over one another [3].
Elastic fibers	Present only in minimal amounts [3].

**Table 2:** Characteristics of the included studies, in chronological order of publication.

Study, Year	Design	*n*	Comparison	Outcomes	Follow-Up
Tabrizi et al., 2020 [31]	RCT (obese patients)	32	PRP vs. CSs	VAS, FFI	8, 12, 24 weeks
Khurana et al., 2021 [32]	RCT	118	PRP vs. CSs	VAS, AOFAS	2, 4, 12, 24 weeks
Kumar N., 2021 [33]	Prospective comparative	50	PRP vs. CSs	VAS, AOFAS	1, 3, 6 months
Vellingiri et al., 2022 [34]	Prospective comparative	110	PRP vs. CSs	VAS, AOFAS	6 months
Alessio-Mazzola et al., 2023 [35]	Retrospective comparative	55	PRP vs. ESWT	VAS, FFI	≥2 years
Pandey et al., 2023 [36]	RCT	72	PRP vs. ESWT	VAS, AOFAS, PFT	15, 30, 90 days
Prakash and Kumar, 2023 [37]	RCT	150	PRP vs. CSs	VAS, AOFAS, PFT	6, 12 weeks, 6 months
Sathyendra et al., 2023 [38]	RCT	70	PRP vs. CSs	VAS	15 days, 1, 3, 6 months
Sharma R. et al., 2023 [39]	RCT	90	PRP vs. CSs	VAS, AOFAS, PFT	6 months
Kamala and Hanumantharaya, 2024 [40]	RCT	40	PRP vs. CSs	VAS, AOFAS	2, 6 weeks, 3, 6 months
Kumar K. et al., 2024 [41]	RCT	70	PRP vs. CSs	VAS, AOFAS	15 days, 1, 3, 6 months
Priyam et al., 2024 [42]	Retrospective comparative	230	PRP vs. CSs	VAS, FAAM	1, 3, 6 months
Sharma G. et al., 2024 [43]	RCT	50	PRP vs. CSs	VAS, AOFAS	3, 6 months
Batra et al., 2025 [44]	RCT	56	PRP vs. CSs	VAS	1, 3, 6 months
Bucak et al., 2025 [45]	Retrospective cohort	152	PRP vs. CSs	VAS, FFI	6 months
Chugh et al., 2025 [46]	RCT	60	PRP vs. CSs	VAS, FFI	4, 12, 24 weeks
Gorantla et al., 2025 [47]	Comparative	80	PRP vs. CSs	VAS, AOFAS, FAAM	6 weeks, 3, 6 months
Patil et al., 2025 [48]	Comparative	90	PRP vs. CSs	VAS, AOFAS	1, 6 weeks, 3, 6 months
Shah et al., 2025 [49]	Prospective interventional	60	PRP vs. CSs	VAS, FAAM	2, 4, 8, 12 weeks
Sunil and Gulati, 2026 [50]	RCT	40	PRP vs. CSs	VAS, AOFAS	1, 3, 6 months
Vyshnavi et al., 2026 [51]	Prospective comparative	120	PRP vs. CSs	VAS, AOFAS, PFT	6, 12 weeks, 6 months
Yadav et al., 2026 [52]	RCT	120	PRP vs. CSs	VAS	6, 12, 24 weeks

AOFAS, American Orthopaedic Foot & Ankle Society score; CSs, corticosteroids; ESWT, extracorporeal shockwave therapy; FAAM, Foot and Ankle Ability Measure; FFI, Foot Function Index; PFT, plantar fascia thickness; PRP, platelet-rich plasma; RCT, randomized controlled trial; VAS, visual analog scale.

**Table 3:** Symptom duration criteria and intervention protocols of the included studies.

Study, Year	Symptom Duration (Inclusion)	PRP Protocol	Comparator Protocol (CS or ESWT)
Tabrizi et al., 2020 [31]	Failed conservative treatment ≥2 months	10 mL blood; single spin (1500 rpm 5 min; ACP Double Syringe System); 3 mL; 3 weekly injections; landmark guided; activation NR	Methylprednisolone 40 mg + 1–1.5 mL lidocaine 1%; single injection
Khurana et al., 2021 [32]	Heel pain >4 weeks	10 mL blood; single spin (1800 rpm 8 min); 2 mL; single injection; peppering technique; no image guidance reported	Methylprednisolone acetate 40 mg (Depo-Medrol, 2 mL) + 1 mL lidocaine 2%
Kumar N., 2021 [33]	≥3 months	27 mL blood; single spin (3200 rpm 12 min); 3 mL; single injection; peppering technique; no image guidance reported	Methylprednisolone 40 mg (2 mL)
Vellingiri et al., 2022 [34]	>3 months	27 mL blood; double spin (1300 rpm 10 min; 3500 rpm 10 min); 3 mL obtained; injected at point of maximum tenderness; activation and image guidance NR	Methylprednisolone 40 mg (2 mL)
Alessio-Mazzola et al., 2023 [35]	>3 months	45 mL blood; triple spin (3550 rpm 12 min; 1100 rpm 10 min; 2600 rpm 20 min); 3 mL; single ultrasound-guided injection	Focal ESWT, ultrasound guided; 4 Hz; 0.03–0.07 mJ/mm^2^, increased to 0.08–0.13 mJ/mm^2^ if tolerated; 1000 impulses per session
Pandey et al., 2023 [36]	>2 months	20 mL blood; double spin (1200 rpm 12 min; 3300 rpm 7 min); 3 mL; single ultrasound-guided injection; activation NR	ESWT: 1 session, 2000 pulses, 0.2 mJ/mm^2^, no local anesthetic
Prakash and Kumar, 2023 [37]	>3 months	3 mL; preparation NR	Methylprednisolone acetate 3 mL
Sathyendra et al., 2023 [38]	>3 months	20 mL blood + 5 mL acid citrate dextrose; double spin (1300 rpm 10 min; 3500 rpm 10 min); 3 mL; single injection	Methylprednisolone acetate 1 mL + 1 mL lidocaine 2%
Sharma R. et al., 2023 [39]	Heel pain >6 weeks	30 mL blood; double spin (2000 rpm; 3000 rpm); palpation guided; volume, activation and number of injections NR	Methylprednisolone 80 mg (2 mL) + 1 mL lidocaine 0.25%; palpation guided
Kamala and Hanumantharaya, 2024 [40]	Not stated	20 mL blood; double spin (1200 rpm 10 min; 2000 rpm 10 min); 2–4 mL obtained	Methylprednisolone acetate 2 mL + 0.5 mL lidocaine 2%
Kumar K. et al., 2024 [41]	Failed conservative treatment ≥3 months	20 mL blood; double spin (1300 rpm 10 min; 3500 rpm 10 min); 3 mL obtained; peppering technique at most tender point; activation and image guidance NR	Methylprednisolone 40 mg + 2 mL lidocaine 2%
Priyam et al., 2024 [42]	>8 months	Not stated	Not stated
Sharma G. et al., 2024 [43]	>6 months	3 mL; preparation NR	Methylprednisolone 40 mg (volume NR)
Batra et al., 2025 [44]	Not stated (mean ≈ 8 months)	NR	Methylprednisolone 40 mg (1 mL); single injection
Bucak et al., 2025 [45]	>3 months	30 mL blood; single spin (3200 rpm 10 min); 3 mL obtained	Triamcinolone acetonide 40 mg (volume NR) + 1 mL lidocaine 2%
Chugh et al., 2025 [46]	>3 months	20 mL blood; single spin (1200 rpm 12 min); 3 mL obtained	Methylprednisolone 40 mg (volume NR)
Gorantla et al., 2025 [47]	Heel pain >6 weeks	Double spin (1200 rpm 10 min; 2400 rpm 10 min); ≈1 mL per tender site after peppering; palpation guided; activation NR	Methylprednisolone (Depo-Medrol) 2 mL + 0.5 mL lidocaine 2%
Patil et al., 2025 [48]	>6 weeks	30 mL blood; single spin (1200 rpm 10 min); 3 mL; single injection	Methylprednisolone acetate (Depo-Medrol) 2 mL + 0.5 mL lidocaine (Xylocaine) 2%
Shah et al., 2025 [49]	Not stated	Not stated	Methylprednisolone 40 mg (volume NR)
Sunil and Gulati, 2026 [50]	>3 months	10 mL blood; single spin (1200 rpm 7 min); 3 mL; single ultrasound-guided injection	Methylprednisolone 40 mg (volume NR)
Vyshnavi et al., 2026 [51]	>6 months	20 mL blood; double spin (3000 rpm 3 min; 4500 rpm 15 min); 4–5 mL; single injection	Methylprednisolone (dose and volume NR) + local anesthetic
Yadav et al., 2026 [52]	Failed conservative treatment ≥6 weeks (>6 months in 60–70%)	20 mL blood; double spin (1500 rpm 10 min; 3000 rpm 15 min); ≈3.5 mL (3–5× baseline platelets); palpation guided; activation NR	Methylprednisolone 80 mg (2 mL) + 4 mL saline + lidocaine 1%

CSs, corticosteroids; ESWT, extracorporeal shockwave therapy; NR, not reported in the original article; PRP, platelet-rich plasma.

**Table 4:** Direction of the between-group differences in pain according to follow-up period.

Follow-Up	Favors CSs or ESWT	No Significant Difference	Favors PRP
Short term (≤6 weeks)	CSs: Prakash [37], Yadav [52]. ESWT: Pandey (day 15) [36]	Sathyendra [38], Kumar K. [41] (15 days); vs. ESWT: Pandey (day 30) [36]	Khurana [32], Shah [49] (from 2 weeks); Sathyendra [38], Kumar K. [41] (1 month)
Medium term (>6 weeks to <6 months)	CSs: Prakash [37], Yadav [52] (12 weeks)	—	Khurana [32], Sathyendra [38], Kumar K. [41], Kamala [40], Shah [49]; vs. ESWT: Pandey (day 90) [36]
Long term (≥6 months)	CSs: Tabrizi (obese patients) [31]	Kumar N. [33]; vs. ESWT: Alessio-Mazzola (≥2 years) [35]	Khurana [32], Prakash [37], Sharma R. [39], Yadav [52], Priyam [42], Vellingiri [34], Sathyendra [38], Kumar K. [41], Kamala [40] and eight further studies [43,44,45,46,47,48,50,51]

Short term, ≤6 weeks; medium term, >6 weeks to <6 months; long term, ≥6 months. CSs, corticosteroids; ESWT, extracorporeal shockwave therapy; FFI, Foot Function Index; PRP, platelet-rich plasma.

**Table 5:** Summary of findings and certainty of the evidence (GRADE).

Outcome (Follow-Up)	Studies (Design)	Direction of Effect	Reasons for Downgrading	Certainty
PRP vs. CSs: pain, short term (≤6 weeks)	6 (5 RCTs, 1 non-randomized)	Inconsistent: 2 favor CSs; 2 no difference at 15 days but favor PRP at 1 month; 2 favor PRP from 2 weeks	Risk of bias (serious); inconsistency (very serious); imprecision (serious)	⊕◯◯◯ Very low
PRP vs. CSs: pain, medium term	7 (6 RCTs, 1 non-randomized)	Favors PRP in 5 of 7 studies; favors CSs in 2 (at 12 weeks)	Risk of bias (serious); inconsistency (serious); imprecision (serious)	⊕◯◯◯ Very low
PRP vs. CSs: pain, long term (≥6 months)	19 (12 RCTs, 7 non-randomized)	Favors PRP in 17 of 19 studies; no difference in 1; favors CSs in 1 (obese patients)	Risk of bias (serious); publication bias (suspected)	⊕⊕◯◯ Low
PRP vs. CSs: function (≥3 months)	6 (5 RCTs, 1 non-randomized)	Favors PRP in 5 studies; favors CSs in 1 (obese patients)	Risk of bias (serious); inconsistency (serious); imprecision (serious)	⊕◯◯◯ Very low
PRP vs. CSs: PFT (≥3 months)	2 RCTs	Greater reduction with PRP	Risk of bias (serious); imprecision (serious)	⊕⊕◯◯ Low
PRP vs. CSs: adverse events	1 non-randomized	Fewer local complications with PRP	Non-randomized design; imprecision (very serious)	⊕◯◯◯ Very low
PRP vs. ESWT: pain (all follow-up periods)	2 (1 RCT, 1 retrospective)	Mixed: ESWT better at day 15, PRP better at day 90, no difference at ≥2 years	Risk of bias (serious); inconsistency (serious); imprecision (very serious)	⊕◯◯◯ Very low

## Data Availability

The study protocol was registered retrospectively in OSF Registries (https://osf.io/hdcjb, accessed on 20 September 2026). Search results, screening decisions and the data extraction spreadsheet will be made available in the associated OSF project upon publication of this manuscript.

## Supplementary Materials

The following supporting information can be downloaded at: https://www.mdpi.com/article/10.3390/ijms27219550/s1.

## Abbreviations

The following abbreviations are used in this manuscript:

AOFAS	American Orthopaedic Foot & Ankle Society score
CI	Confidence interval
CSs	Corticosteroids
EGF	Epidermal growth factor
ESWT	Extracorporeal shockwave therapy
FAAM	Foot and Ankle Ability Measure
FFI	Foot Function Index
FGF	Fibroblast growth factor
GRADE	Grading of Recommendations Assessment, Development and Evaluation
HGF	Hepatocyte growth factor
IGF-1	Insulin-like growth factor 1
MeSH	Medical Subject Headings
NR	Not reported
OSF	Open Science Framework
PDGF	Platelet-derived growth factor
PFT	Plantar fascia thickness
PICOS	Population, Intervention, Comparator, Outcomes and Study design
PRISMA	Preferred Reporting Items for Systematic Reviews and Meta-Analyses
PRP	Platelet-rich plasma
RCT	Randomized controlled trial
SDF-1α	Stromal cell-derived factor 1α
TGF-β	Transforming growth factor β
VAS	Visual analog scale
