# Supplementary material for: Efficacy of Platelet-Rich Plasma in the Treatment of Chronic Plantar Fasciitis Compared with Other Conventional Treatments (Corticosteroids and Shockwave Therapy): A Systematic Review
Source: Int J Mol Sci. 2026 Oct 26;27(21):9550. doi: 10.3390/ijms27219550 (PMC13762751; doi:10.3390/ijms27219550)
Supplement: Supplementary file 1 [file ijms-27-09550-s001.zip › ijms-4615584-supplementary.pdf]

Supplementary Materials

Efficacy of Platelet-Rich Plasma in the Treatment of Chronic Plantar Fasciitis Compared with Other Conventional Treatments (Corticosteroids and Shockwave Therapy): A Systematic Review

Mercedes Ortiz-Romero, Elías Parra-Aguado and Luis M. Gordillo-Fernández

Table S1. Full search strategy for each database, with the search date, limits applied and number of records retrieved.

Database	Search date	Search string	Limits	Records retrieved
PubMed/MEDLINE	September 2026	("Fasciitis, Plantar" OR "plantar fasciitis" OR "plantar fasciopathy" OR "plantar fasciosis" OR "heel pain") AND ("Platelet-Rich Plasma" OR "platelet-rich plasma" OR "platelet rich plasma" OR PRP) AND ("Adrenal Cortex Hormones" OR "Glucocorticoids" OR corticosteroid* OR steroid* OR "Extracorporeal Shockwave Therapy" OR "shock wave" OR shockwave OR ESWT)	Publication date: 2020/01/01 to search date	74
Scopus	September 2026	("plantar fasciitis" OR "plantar fasciopathy" OR "plantar fasciosis" OR "heel pain") AND ("platelet-rich plasma" OR "platelet rich plasma" OR PRP) AND (corticosteroid* OR steroid* OR "shock wave" OR shockwave OR ESWT)	PUBYEAR > 2019	100
Web of Science Core Collection	September 2026	("plantar fasciitis" OR "plantar fasciopathy" OR "plantar fasciosis" OR "heel pain") AND ("platelet-rich plasma" OR "platelet rich plasma" OR PRP) AND (corticosteroid* OR steroid* OR "shock wave" OR shockwave OR ESWT)	Publication years: 2020–2026	103
Cochrane Library (CENTRAL)	September 2026	#1 MeSH descriptor: [Fasciitis, Plantar] explode all trees<br>#2 ("plantar fasciitis" OR "plantar fasciopathy" OR "plantar fasciosis" OR "heel pain"):ti,ab,kw<br>#3 #1 OR #2<br>#4 MeSH descriptor: [Platelet-Rich Plasma] explode all trees<br>#5 ("platelet-rich plasma" OR "platelet rich plasma" OR PRP):ti,ab,kw<br>#6 #4 OR #5<br>#7 MeSH descriptor: [Adrenal Cortex Hormones] explode all trees<br>#8 MeSH descriptor: [Extracorporeal Shockwave Therapy] explode all trees<br>#9 (corticosteroid* OR steroid* OR "shock wave" OR shockwave OR ESWT):ti,ab,kw<br>#10 #7 OR #8 OR #9<br>#11 #3 AND #6 AND #10	Publication date: January 2020 to September 2026	69
Dialnet	September 2026	"plasma rico en plaquetas" AND "fascitis plantar"<br>"platelet-rich plasma" AND "plantar fasciitis"	Publication years: 2020–2026	9

**Total**	**355**

Duplicates (n = 148) were removed before screening, leaving 207 records. The search strings followed a PICOS-based structure combining controlled vocabulary (MeSH) and free-text terms for the condition, platelet-rich plasma and the comparators (corticosteroids and extracorporeal shockwave therapy).
